# Pharmacological Activities, Therapeutic Effects, and Mechanistic Actions of Trigonelline

**DOI:** 10.3390/ijms25063385

**Published:** 2024-03-16

**Authors:** Vi Nguyen, Elaine G. Taine, Dehao Meng, Taixing Cui, Wenbin Tan

**Affiliations:** 1 Department of Cell Biology and Anatomy, School of Medicine, University of South Carolina, Columbia, SC 29209, USA; vi.nguyen@uscmed.sc.edu; 2 TritaliMed, Inc., Columbia, SC 29223, USA; 3 Applied Physics Program, California State University San Marcos, San Marcos, CA 92096, USA; 4 Dalton Cardiovascular Research Center, Department of Medical Pharmacology and Physiology, School of Medicine, University of Missouri, Columbia, MO 65211, USA; taixingcui@health.missouri.edu; 5 Department of Biomedical Engineering, College of Engineering and Computing, University of South Carolina, Columbia, SC 29208, USA

**Keywords:** trigonelline, inflammation, oxidation, metabolic homeostasis, neuromodulation, chronic metabolic diseases

## Abstract

Trigonelline (TRG) is a natural polar hydrophilic alkaloid that is found in many plants such as green coffee beans and fenugreek seeds. TRG potentially acts on multiple molecular targets, including nuclear factor erythroid 2-related factor 2 (Nrf2), peroxisome proliferator-activated receptor γ, glycogen synthase kinase, tyrosinase, nerve growth factor, estrogen receptor, amyloid-β peptide, and several neurotransmitter receptors. In this review, we systematically summarize the pharmacological activities, medicinal properties, and mechanistic actions of TRG as a potential therapeutic agent. Mechanistically, TRG can facilitate the maintenance and restoration of the metabolic homeostasis of glucose and lipids. It can counteract inflammatory constituents at multiple levels by hampering pro-inflammatory factor release, alleviating inflammatory propagation, and attenuating tissue injury. It concurrently modulates oxidative stress by the blockage of the detrimental Nrf2 pathway when autophagy is impaired. Therefore, it exerts diverse therapeutic effects on a variety of pathological conditions associated with chronic metabolic diseases and age-related disorders. It shows multidimensional effects, including neuroprotection from neurodegenerative disorders and diabetic peripheral neuropathy, neuromodulation, mitigation of cardiovascular disorders, skin diseases, diabetic mellitus, liver and kidney injuries, and anti-pathogen and anti-tumor activities. Further validations are required to define its specific targeting molecules, dissect the underlying mechanistic networks, and corroborate its efficacy in clinical trials.

## 1. Introduction

**Figure 1:**
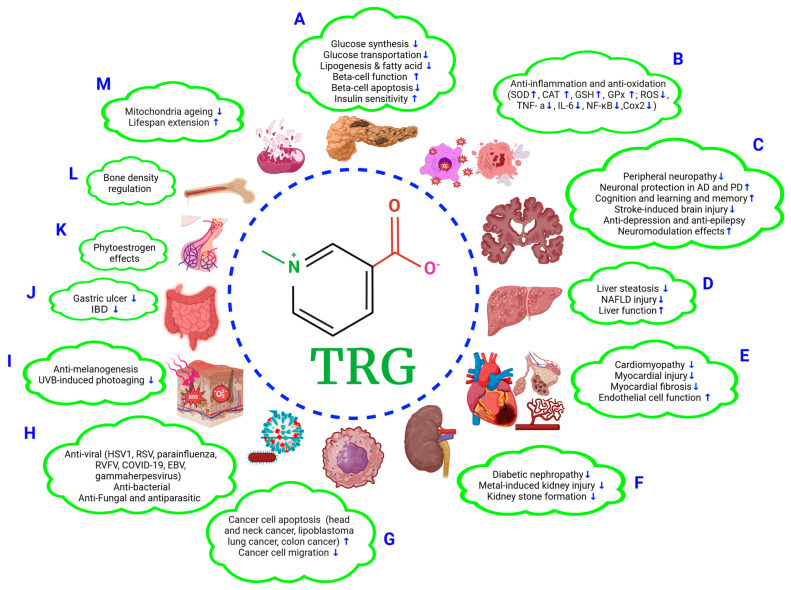
A summary of therapeutic effects of TRG on multiple organs. TRG shows various beneficial roles in many pathological conditions. It can (1) modulate glucose and lipid homeostasis [4,5,6,7,8,9,10] (**A**); (2) suppress the inflammatory response and oxidative stress [11,12,13,14] (**B**,**H**,**M**); (2) facilitate recovery from neurological impairments such as neurodegenerative disorders [15,16,17,18], ischemia-induced brain damage [19,20], cognitive decline [21,22,23], diabetic peripheral neuropathy [24,25], depression, and epilepsy [26,27,28] (**C**); (3) mitigate DM and its complications [6,11,29,30,31,32] (**A**–**F**); (4) alleviate cellular injuries in the cardiovascular system [33,34], liver [35,36], kidney [37,38,39,40,41,42], gastric system [43,44], and skin [45,46,47] (**D**–**F**,**I**–**L**); and (5) inhibit proliferation and migration of tumor cells [48,49,50] (**G**). ↑, Increasing; ↓, decreasing. The graph was created with Biorender.com (accessed on 29 December 2023).

**Figure 2:**
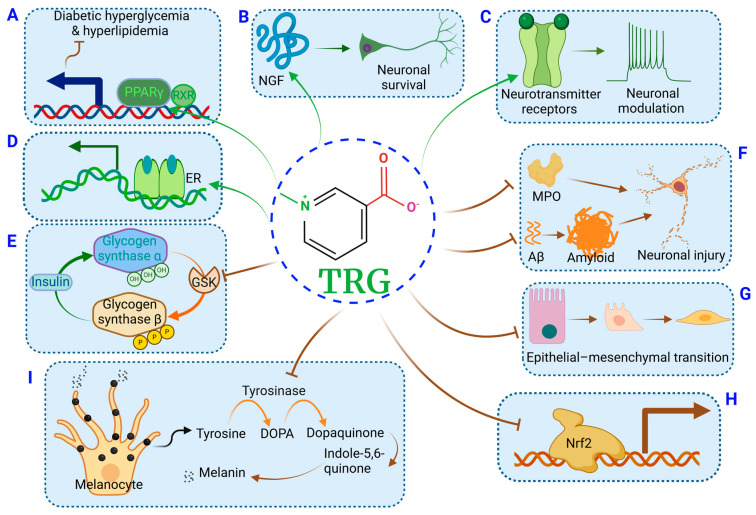
The proposed mechanistic actions of TRG on multiple targets. TRG can potentially bind to PPARγ, NGF, and several neurotransmitter receptors, restoring glucose and lipid homeostasis, promoting neuronal survival, and modulating neuronal activity [25,30,51] (**A**–**C**). TRG can act as a phytoestrogen to stimulate ER [52] (**D**). TRG can potentially bind to GSK (**E**), MPO, and Aβ (**F**), executing inhibitory effects [7,15,19]. TRG can prevent EMT (**G**), interfere with Nrf2 nuclear translocation (**H**), and suppress tyrosinase activity (**I**) [37,38,53,54,55]. The graph was created with Biorender.com (accessed on 29 December 2023).

Trigonelline (TRG) was first isolated by Johns E. from fenugreek (*Trigonella foenum-graecum*) in 1885 [1]. It is synthesized by the methylation of nicotinic acid (niacin or vitamin B3) and, thus, it is also called N-methyl nicotinic acid. *S*-adenosyl-l-methionine provides a methyl group for TRG synthesis. TRG exerts its function as a plant hormone, a dietary ingredient, and a urinary metabolite in humans. In plants, TRG executes various functions including plant cell cycle modulation and nodulation for plant growth and survival. TRG is abundant in coffee beans, accounting for 1–3% of their dry weights. As a dietary substance, TRG can be demethylated to nicotinic acid and also yield 1-methylpyridinium and 1,2-dimethylpyridinium during roasting [2,3]. Within the past few decades, studies on TRG extensively revealed its multi-targeting therapeutic effects in various pathological conditions encompassing metabolic syndrome, neurodegenerative diseases, cancers, and inflammation-associated disorders (Figure 1). TRG can potentially target multiple molecules, such as peroxisome proliferator-activated receptor γ (PPARγ), glycogen synthase kinase (GSK), tyrosinase, nerve growth factor (NGF), amyloid-β peptide (Aβ), estrogen receptor, and several neurotransmitter receptors (Figure 2). The hubs of the biological activities of TRG are glucose and lipid metabolic regulation and inflammatory and oxidative stress modulation, which result in homeostasis restoration and protective actions in many organs and tissues.

### 1.1. Safety Profile of TRG

In acute oral toxicity studies, no mortality and changes in behavior were observed in TRG-treated mice up to the dose of 5000 mg/kg. TRG (75 mg/kg, p.o.) does not affect the estrous cycle of rats and showed no effect on fertility in pregnant and non-pregnant female rats, with no fetal miscarriage or deformities [56]. A fetotoxicity study showed that the standardized fenugreek seed extracts IDM01 (containing 28.8% of 4-hydroxyisoleucine and 34.8% of TRG, administered by oral gavage at a dose of 500 mg/kg/day) had no adverse effects in pregnant rats on maternal parameters and fetal development [57]. According to the National Cancer Institute (NCI) guidelines, compounds are considered cytotoxic when their inhibitory concentration 50% (IC50) values are less than 20–30 µg/mL. Normal lung cells treated with 50 µM (6.86 µg/mL) TRG alone did not exhibit cytotoxicity [58]. In a risk assessment study of the effects of TRG from coffee and coffee by-products on human health, no evidence of adverse effects was found after acute exposure. The ingestion of TRG as a component of coffee or coffee-related products was shown to be safe, since the long-term traditional consumption of these products exhibits a good safety profile for human health [59].

### 1.2. Pharmacokinetics of TRG

When TRG was administered by oral gavage (p.o., 10 mg/kg) or intravenously (i.v., 5 mg/kg) to Sprague Dawley rats, the AUC (0–infinity; area under the plasma concentration–time curve) values were 4.1 ± 1.2 (p.o.) and 3.5 ± 0.9 (i.v.) min.mg/mL. The t1/2b (biological half-life) was 3.6 ± 0.3 (p.o.) and 3.4 ± 0.6 (i.v.) h. The CL (clearance) values were 0.9 ± 0.2 (p.o.) and 0.9 ± 0.2 (i.v.) mL/min. After oral administration, C*_max_* and T*_max_* of TRG were 12.3 ± 2.9 mg/L and 1.2 ± 0.4 h, respectively [60]. In a New Zealand white rabbit model, when TRG was administered by oral gavage (p.o., 10 mg/kg), the pharmacokinetic parameters were 0.121 mg/mL (C*_max_*), 1.30 h (T*_max_*), 20.06 h (t1/2b), 7.59 µg/mL·h (AUC^0–24^), and 1.32 L/h (CL) [61].

## 2. Regulatory Role of TRG in Glucose and Lipid Metabolism

### 2.1. TRG Regulates Glucose Synthesis and Transport (Figure 1A)

TRG reduced the early glucose and insulin responses during an oral glucose tolerance test (OGTT). In a 2 h OGTT in overweight men (n = 15), TRG ingestion reduced glucose and insulin concentrations after 15 min compared with a placebo [62]. In type 2 diabetic Goto–Kakizaki (GK) rats, TRG decreased the expression of genes involved in glycolysis (*Pdhb*, *Pklr*, *Pfkfb1*, and *Gck*), gluconeogenesis (*G6pc*, *Slc37a4*), and glucose uptake (*Slc2a2*) [4]. TRG inhibited the expression of glucose transporter 4 (*Glut4*) in adipocytes [5].

### 2.2. TRG Modulates Lipogenesis and Fatty Acid Metabolism (Figure 1A)

TRG was shown to regulate lipid metabolism enzymes in non-obese type 2 diabetic GK rats. TRG increased the activity of liver fatty acid synthase (FAS), liver carnitine palmitoyl transferase (CPT), and glucokinase (GLK), and decreased the serum and liver triglyceride (TG) levels, suggesting that it could suppress both TG accumulation and the progression of diabetes through the regulation of these enzymes’ activities [63]. In type 2 diabetic GK rats, TRG downregulated the lipid metabolism by reducing the expression of genes involving cholesterol biosynthesis (*Lss*, *Hmgcr*, *Sqle*, *Fdft1*, *Cyp51*, and *Sc4mol*) and fatty acid metabolism (*Acsl5*, *Acacb*, *Acaca*, and *Srebf1*). TRG attenuated necrosis factor-alpha (TNF-α) signaling by decreasing the expression of the *TNF-α* receptor gene [4]. TRG suppressed the accumulation of lipid droplets within adipocytes via downregulating the expression of PPARγ and C/EBP-α, leading to a decrease in the expression levels of many lipid-related genes such as *adiponectin*, *adipogenin*, *leptin*, *resistin*, *FAS*, and adipocyte fatty acid-binding protein in 3T3-L1 cells [5]. TRG might compete against the agonist troglitazone for binding to PPARγ [5]. TRG induced the browning of 3T3-L1 white adipocytes by activating β3-AR and inhibiting PDE4, thereby stimulating the p38 MAPK/ATF-2 signaling pathway. TRG was shown to decrease adipogenesis and lipogenesis, facilitate the oxidation and lipolysis of fatty acids, and upregulate the expressions of transcripts related to mitochondrial biogenetics such as *Cox4*, *Nrf1*, and *Tfam* in white adipocytes, thus exerting anti-obesity functions [64].

## 3. Anti-Diabetic Mellitus (DM) Effects of TRG

### 3.1. Improvements in β-Cell Function and Mitigation of β-Cell Apoptosis (Figure 1A)

In streptozotocin (STZ)-induced diabetic mice, TRG decreased the levels of blood glucose, serum TNF-α, interleukin 6 (IL-6), IL-1β, and malondialdehyde (MAD); it increased the levels of serum insulin and adiponectin and pancreatic glutathione (GSH), superoxide dismutase (SOD), and catalase (CAT) activities. Furthermore, TRG decreased caspase-3 expression in β-cells and inhibited β-cell apoptosis [29]. In type 2 DM (T2DM) rats, TRG improved serum parameters (such as FFA, TNF-α, IL-6, and leptin), decreased the levels of apoptotic ER stress markers (CHOP, caspase-12, and caspase-3), and enhanced PPARγ expression in the adipose tissue [30]. A molecular docking assay indicated the potential binding of TRG with PPRAγ. TRG forms C=O···π and C–H···π interactions with G284 and I341 of PPARγ with a contact distance of 4.3 and 3.8 Å, respectively. The Glide energy is about −17.636 kcal/mol. The residues R288 (*P_pKa_* = 14.54), C285 (*P_pKa_* = 11.25), G284, I281, M348, I341, and S342 of PPARγ also form Van der Waals (VdW) interactions in contact with TRG [30] (Figure 2A). TRG was shown to exert an insulin-sensitizing effect by alleviating ER and oxidative stress in the pancreas [30]. In alloxan-induced diabetic rats, TRG could inhibit the activities of dipeptidyl peptidase-4 (DPP-4) and alpha-glucosidase and facilitate the secretion of insulin by β-cells by stimulating the action of glucagon-like peptide-1 (GLP-1). TRG appeared to decrease the activities of intestinal maltase, lactase, and sucrase, modulate the activity of ACE in serum and kidney, restore the functions of the liver and the serum lipid profiles, decrease the glycosylated hemoglobin levels, and ameliorate damage in pancreatic islets and β-cells [31]. In diabetic pregnant mice, TRG reduced β-cell mass and apoptosis and the levels of placental proinflammatory cytokines and promoted β-cell replication. TRG reversed diabetic-related hyperglycemia, dyslipidemia, insulin resistance, adipocytokine dysregulation, and the drop of fetus number, fetal weight, and the fetal/placental ratio in diabetic pregnant mice. Therefore, TRG can suppress inflammation, regulate the secretion of adipocytokines, and protect β-cell functions in diabetic pregnant mice [32].

### 3.2. Effects on Oxidative Stress (Figure 1A,B)

In STZ-induced and high-carbohydrate/high-fat diet (HFD)-fed rats, TRG increased the activities of antioxidant enzymes by elevating the SOD, CAT, and GSH levels [11]. TRG restored the levels of blood glucose, total cholesterol (TC), and TG, the pancreas-to-body weight ratio, insulin secretion, the insulin sensitivity index, the insulin content in the pancreas, and the MAD and nitric oxide (NO) content to near-normal levels in diabetic rats [11]. TRG-containing fenugreek seed extracts decreased the levels of oxidative stress markers such as MDA and increased the plasma GSH levels in diabetic rabbits [12]. In STZ-induced HFD-fed T2DM rats, TRG induced the expression of PPAR-γ and inhibited GLUT4 and TNF-α. TRG decreased blood sugar levels, inflammatory response, and oxidative stress and improved kidney function by decreasing blood urea nitrogen (BUN) and creatinine levels in T2DM rats [13]. TRG-enriched extracts of *Trigonella stellata* were shown to decrease the blood glucose level and recover the biochemical markers of both liver (transaminase activities, lactate dehydrogenase and gamma-glutamyl transferase levels) and renal function (BUN, creatinine, and bilirubin levels) in diabetic rats. They could increase the activity of CAT, SOD, and GSH by restoring the levels of glutathione peroxidase (GPx), CYP2E, CYP3A4, glutathione S-transferase (GST), and glutathione reductase (GR) in diabetic rats [14].

### 3.3. Hypoglycemic Effect

TRG showed anti-diabetic effects in both nonobese and obese T2DM animal models by decreasing the levels of blood glucose, insulin, TNF-α, HbA_1c_, TC, LDL-c, TG, FFA, etc. [65] TRG could decrease the activities of intestinal a-amylase, maltase, and lipase by 36%, 52%, and 56%, respectively [6]. It reduced the blood glucose level by 46%. It improved glucose, maltase, starch, and lipid oral tolerance and protected the liver and kidney functions in a diabetic rat model [6]. GSK-3a and 3b were shown to negatively regulate glycogen synthase activity and insulin sensitivity [66]. A molecular docking study showed that the binding energy of TRG to GSK-3a and GSK-3b is −6.85 and −5.51 Kcal/Mol, respectively, suggesting that TRG has the potential to act as an inhibitor of GSK to maintain normoglycemia [7] (Figure 2E). The oral administration of the compound GTF-231 (a combination of gymnemic acid, TRG, and ferulic acid in the ratio of 2:3:1) improved glucose homeostasis and showed antioxidant properties in the pancreatic tissue in the low-dose STZ-induced HFD-fed T2DM rat model [8]. Dietary TRG improved the diabetic condition by suppressing internal oxide generation and modulated the hepatic expression of diabetes-related genes in nonobese T2DM rats [65].

In the T2DM KK-A^y^ obese mouse model, TRG reduced the plasma levels of glucose and insulin and improved glucose tolerance and the insulin resistance index calculated by the homeostasis model assessment of insulin resistance (HOMA-IR) [9]. HFD-fed mice administered a TRG-enriched yogurt showed decreases in the levels of fat accumulation, advanced glycation end products (AGEs), and AGE receptors and increases in the levels of insulin sensitivity, glucose tolerance, and components related to AGE detoxification [10]. TRG was shown to exert anti-inflammatory and antiglycation activities in the liver and kidney [10]. In addition, the serum TRG levels were associated with a decrease in the HbA_1c_ levels over time in a biomarker analysis of glucose homeostasis in 3986 participants at both baseline and a 5-year follow-up [67].

### 3.4. Improvements in Insulin Sensitivity (Figure 1A)

TRG exhibited an insulin-sensitizing effect in STZ-induced HFD- fed diabetic rats by reducing the HOMA-IR index. TRG attenuated the elevated serum levels of glucose, glycosylated hemoglobin, AST, ALT, ALP, and lipids and improved the insulin level in insulin-resistant diabetic rats [68]. TRG decreased the levels of serum glucose and fructosamine and the HOMA-IR index. It improved insulin sensitivity in the soleus muscle of T2DM rats by increasing insulin receptor autophosphorylation and the levels of its downstream effectors such as phosphorylated Akt (T308) and GLUT4, thus modulating glucose uptake [69]. Patients with DM who received 2 g of TRG-containing fenugreek per day for 12 weeks exhibited a higher level of fasting insulin and a lower ratio of high-density lipoproteins to low-density lipoproteins [70].

## 4. Neuroprotective Effects of TRG

### 4.1. Anti-Diabetic Peripheral Neuropathy (Figure 1C)

TRG showed beneficial effects against diabetic peripheral neuropathy. In diabetic rats, TRG restored the altered levels of serum glucose, serum insulin, insulin sensitivity index, and lipid peroxidation, micropathological aberrances of the sciatic nerve, abnormalities in body weight, sciatic nerve conduction velocity, nociception, antioxidant enzyme activity, GLP-1 receptor mRNA and protein levels, total and phosphorylated p38 mitogen-activated protein kinase (MAPK) levels, MDA content, and SOD activity [24]. TRG (2.5 or 5 mg/mL) increased the NGF levels in spiral ganglions. It reduced auditory damage caused by diabetic progression in LepR(db/db) mice. Further molecular docking simulations indicated that TRG could bind to the active site of NGF through interactions with lysine 88 (Lys88) and tyrosine 52 (Tyr52) [25] (Figure 2B).

### 4.2. Effects on Alzheimer’s Disease (AD) (Figure 1C)

TRG could lower the occurrence of AD by reducing Aβ accumulation and neurotoxicity. TRG showed a relatively high binding affinity for Aβ and could alter its structure, inhibiting its aggregation [15] (Figure 2F). TRG was found in the cerebral cortex after oral administration (10 mg/kg/day), indicating that it might be able to pass through the blood–brain barrier. It was shown to reverse Aβ-induced axonal atrophy by binding and activating creatine kinase B-type (CKB) [16]. It also improved the memories of object recognition and object location and restored the levels of the neurofilament light chain in the cerebral cortex [16]. TRG appeared to improve cognition by alleviating hippocampal neuronal injury induced by intracerebral Aβ (1–40). It could suppress oxidative stress, astrocyte activity, and inflammation by decreasing the levels of glial fibrillary acidic protein (GFAP), S100b, cyclooxygenase 2 (Cox2), TNF-α, and IL-6, with no significant alteration in inducible NO synthase (iNOS), and preserve mitochondrial integrity by improving mitochondrial membrane potential (MtMP) and GSH and SOD expression [17].

### 4.3. Effects on Parkinson’s Disease (PD) (Figure 1C)

In a 6-hydroxydopamine (6-OHDA)-induced rat model of PD, TRG (50 and 100 mg/kg) increased the viability of substantia nigra pars compacta neurons, prevented apoptosis, and decreased the MDA levels, thereby reducing the neurodegenerative effects of the neurotoxin 6-OHDA [18].

### 4.4. Effects on Cognition, Learning, and Memory (Figure 1C)

TRG alleviated lipopolysaccharide (LPS)-induced cognitive dysfunctions through suppressing the TLR4/NF-kB pathway, decreasing hippocampal MDA and acetylcholinesterase (AChE) activity, and increasing SOD, CAT, and GSH levels [71]. TRG decreased LPS-induced elevation in TNF-α, IL-6, oxidative stress, and AchE levels in both the hippocampus and the cortex. It restored the LPS-depleted brain-derived neurotrophic factor (BDNF) levels and reversed LPS-mediated cognitive impairment and behavioral disturbance [21]. TRG (50 and 100 mg/kg) treatment improved cognitive performance by lowering the AGE and AChE levels and reversing oxidative damage in a D-galactose (D-gal)-induced amnesia model [22]. TRG ameliorated learning and memory decline in the senescence-accelerated mouse-prone 8 (SAMP8) model [23]. Particularly, TRG exerted effects in a wide range of biological processes including nervous system development, mitochondrial function, ATP synthesis, inflammation, autophagy, and neurotransmitter release through negatively modulating Traf6-mediated NF-kB activation in the SAMP8 model. TRG suppressed the expression of proinflammatory cytokines such as TNF-α and IL6 and enhanced the release of neurotransmitters including dopamine, noradrenaline, and serotonin in the mouse hippocampus [23].

### 4.5. Effects on Stroke (Figure 1C)

Myeloperoxidase (MPO) plays an important role in stroke-related inflammation [72]. TRG (100 mg/kg, i.p.) could suppress the elevated levels of nitrite and MDA post-stroke, increase the GSH levels, and reduce cerebral infarction with an improvement in motor and neurodeficit scores. TRG interacted with MPO by forming hydrogen bonds with N-acetylglucosamine (NAG), arginine (ARG), and tryptophan (TRP), thus conferring brain protection from MPO-mediated inflammation after stroke [19] (Figure 2F). In an oxygen-glucose deprivation/reperfusion (OGD/R) model, TRG reversed the injury-induced decreased levels of SOD and GPx and increased levels of TNF-α, IL-6, IL-1β, caspase-3 activity, as well as Bcl-2-associated protein X (Bax) expression, and activated the PI3K/Akt signaling pathway to protect hippocampal neurons [20].

### 4.6. Anti-Depression and Anti-Epilepsy Effects (Figure 1C)

TRG increased the hippocampal CA1 area through attenuating N-methyl-D-aspartate (NMDA) receptor activity, thus exerting an antidepressant-like effect in a depressive-like mouse model [26]. In a maternal separation (MS) stress-induced depressive- and anxiety-like mouse model, TRG reversed the negative behavioral effects caused by MS stress by modulating oxidative stress via decreasing the MAD and NO levels, while increasing brain and serum antioxidant capacity [27]. In the kainic acid-induced epileptic model, the administration of TRG (100 mg/kg, i.p. for 14 days) improved behaviors, e.g., by promoting the amelioration of anxiety and decreasing memory impairment. TRG showed neuroprotective effects, mitigating cytotoxicity and neuronal injury within the hippocampus by reducing the levels of inflammatory cytokines and oxidative stress biomarkers. It showed therapeutic promise for epilepsy management [28].

### 4.7. Neuromodulation (Figure 1C)

TRG has a sedative role [73]. TRG-enriched green coffee bean powders could function as a sleep aid and pain relief (Tan, W. (University of South Carolina, Columbia, MO, USA); Cui, T. (University of Missouri, Columbia, MO, USA). Personal communication, 2023). A molecular docking study showed many potential binding targets of TRG among neurotransmitter receptors, including GABAa receptor, GABAb receptor, M1 mAChR, calcium-sensing receptor, M2 mAChR, 5HT2aR, 5HT1aR, insulin receptor, NMDAR, AMPAR, α7 nAChR, α4β2 nAChR, and D2 and D1 dopamine receptors [51] (Figure 2C). For example, the binding score of TRG to the catalytic site of GABAa receptor is −1.62, while that of its agonist benzamidine is −1.92. The binding score of TRG to the binding pocket of GABAb receptor is −1.84, while that of its agonist (R)-baclofen is −3.03 [51]. This might be a contributing factor to the sedative role of TRG.

## 5. Liver Protection Effects of TRG

### 5.1. Alleviation of Liver Steatosis and NAFLD Injury (Figure 1D)

In high-cholesterol (HC) and HFD-fed C57BL/6J mice, TRG restored hepatic cellular autophagy and reduced lipotoxicity to prevent steatosis by enhancing AMPK and reducing mTOR activity, showing a therapeutic effect against non-alcoholic fatty liver disease (NAFLD) [74]. In an NAFLD rat model, TRG protected the liver cells by enhancing the expression of the apoptosis inhibitor gene *Bcl-2*, while decreasing the expression of *Bax* in the liver tissues, lowering hepatocytic death and reducing hepatic steatosis. TRG reduced the elevated hepatic levels of ALT, AST, TC, LDL-C, and MDA and increased the SOD levels [75].

### 5.2. Improving Liver Function (Figure 1B,D)

In high-fat high-fructose (HFHF) diet-induced insulin resistance (IR) rats, TRG (50 and 100 mg/kg, p.o.) administration decreased the HOMA-IR index and the levels of hepatic lipids, oxidative stress biomarkers, and inflammatory cytokines, exhibiting a protective effect against IR-induced liver complications [35]. A proteomics study in HepG2 cells showed that TRG exerted hepatoprotective effects by upregulating proteins related to a structural constituent of ribosomes, ATPase activity, and ADP binding (such as RPL14, RPL24, and SNRPD) and downregulating cysteine-type endopeptidase activator activity involving the apoptotic process and enzyme binding (such as RPS27L, RPL18A, PDCD5, HNRNPF, RRIA, HSPA8, TUBA1B, GNB2L, and PCBP2) [36].

## 6. Cardiovascular Protection Effects of TRG

### 6.1. Anti-Cardiomyopathy Effect (Figure 1E)

TRG reduced H2O2-induced necrosis and apoptosis in cardiomyocyte-like H9C2 cells by downregulating the expression of the apoptotic genes such as *caspase-3* and *caspase-9* and upregulating the expression of the anti-apoptotic genes *Bcl-2* and *B-cell lymphoma-extra-large* (*Bcl-XL*) [76]. The levels of trimethylamine N-oxide (TMAO) are linked to atherothrombotic cardiovascular disease [77]. TRG inhibited not only trimethylamine (TAM) production by intestinal bacteria but also the activity of the FMO enzyme from converting TAM to TMAO [78].

### 6.2. Mitigation of Myocardial Injury (Figure 1E)

In nicotinamide (NICO)- and STZ-induced DM rats, TRG reduced myocardial necrosis/ischemia and improved cardiac functions by lowering the levels of creatine kinase isoenzyme, LDH, and AST, and restoring the drop of blood pressure caused by myocardial damage [33]. In adult rats with isoproterenol (ISO)-induced myocardial dysfunctions, TRG prevented ISO-induced myocardial injury by downregulating the expression of the proteins Hsp27 and αB-crystallin [34].

### 6.3. Alleviation of Fibrosis (Figure 1B,E)

TRG was shown to prevent fibrosis by suppressing the spontaneous self-assembly of type I collagen in the heart [79]. In bleomycin (BLM)-induced pulmonary fibrosis (PF), TRG mitigated the inflammatory process via targeting NF-κB/NLRP3/IL-1β signaling, activating autophagy, attenuating alveolar epithelial cell apoptosis and senescence, decreasing the activation of the lung SPHK1/S1P axis and the expression of its downstream Hippo targets such as *Yap*/*Taz* profibrotic genes, and reversing epithelial–mesenchymal transition (EMT). Thus, TRG could exert prophylactic and antifibrotic effects against BLM-induced PF [53] (Figure 2G).

### 6.4. Improving Endothelial Function (Figure 1E)

Ingestion of TRG-enriched Sakurajima radish (170 g/day, for 10 days) increased the plasma TRG levels and improved flow-mediated dilation, an index of vascular endothelial function [80].

## 7. Anti-Nephropathy Effects of TRG

### 7.1. Anti-Diabetic Nephropathy (Figure 1F)

The anti-fibrotic effects of TRG in renal tubular epithelial cells were assessed in the diabetic kidney disease (DKD) models. TRG pretreatment could attenuate oxalate-induced EMT features including (1) spindle-shape morphological changes, (2) the elevated expression of mesenchymal markers such as fibronectin, vimentin, MMP9, and α-smooth muscle actin (α-SMA), and (3) the decreased expression of epithelial proteins such as E-cadherin and zonula occludens-1. Moreover, TRG also prevented oxalate-induced cell migration, ROS overproduction, and the downregulation of the nuclear factor erythroid 2-related factor 2 (Nrf-2) signaling [37] (Figure 2H). TRG could alleviate tubular EMT and renal fibrosis in the db/db DKD mouse model by upregulating Smad7 expression in proximal tubule epithelial cells (PTCs) [38] (Figure 2G). In a diabetic nephropathy human mesangial cell (HMC) model, TRG increased miR-5189-5p expression, reduced hypoxia-inducible factor 1 subunit alpha inhibitor (HIF1AN) levels, modulated the AMPK pathway, and promoted autophagy, demonstrating a protective role in response to high glucose exposure [39]. Kidneys from patients with diabetes exhibited higher levels of Nrf2 and SGLT2 in renal PTCs than kidneys from patients without diabetes. In a mouse model of Nrf2 overexpression in renal PTCs, Nrf2 was shown to mediate an increase in blood glucose, glomerular filtration rate, urinary albumin-to-creatinine ratio, tubulointerstitial fibrosis, and sodium–glucose cotransporter 2 (Sglt2) expression, which could be inhibited by TRG or Nrf2 siRNA [81].

In a model of non-insulin-dependent neonatal diabetic rats, TRG decreased early and late apoptotic renal cell death and reduced kidney hypertrophic growth through suppressing TNF-α signaling, playing a role in the alleviation of kidney damage [40]. TRG appeared to regulate the expression of miR-3550 and downregulate the abnormally activated Wnt/b-catenin signaling pathway, thereby reducing the damage caused by diabetic nephropathy in STZ-induced DM rats [41]. TRG relieved renal damage by reducing BUN, creatinine, and albumin levels in T2DM rats [13]. Nrf2 could induce the intrarenal expression of the *Ras* gene, while diabetic hypertension and renal damage could result from chronic hyperglycemia. Genetic deletion of *Nrf2* or TRG inhibition of Nrf2 in diabetic Akita mice attenuated hypertension, renal injury, tubulointerstitial fibrosis, and the urinary albumin/creatinine ratio. In cultured IRPTCs, *Nrf2* siRNA or TRG prevented the high glucose–induced Nrf2 nuclear translocation and expression of angiotensinogen (*Agt*) and angiotensin-converting enzyme (*ACE*), with transcriptional augmentation of *ACE2* and *angiotensin 1–7* (*Ang 1–7*) receptor [42].

### 7.2. Effects on Metal Exposure-Induced Renal Tubular Injury (Figure 1F)

Upon acute iron exposure, Nrf2 was shown to protect kidneys from iron-induced injury, whereas chronic iron exposure could induce oxidative stress and exhaust the antioxidant Nrf2 pathway, leading to renal injury. In human conditionally immortalized PTCs, long-term iron exposure resulted in iron accumulation, cytosolic ROS formation, increases in *heme oxygenase 1* (*HMOX-1*) mRNA expression, nuclear translocation of Nrf2, and induction of *NQO1*. TRG could suppress iron-induced ROS production, thus protecting from renal injury [82].

### 7.3. Preventive Effect against Kidney Stone Formation (Figure 1F)

TRG was shown to reduce calcium oxalate monohydrate (COM) crystal size, number, and mass during crystallization by inhibiting crystal growth and crystal–cell adhesion but not crystal aggregation. TRG decreased the level of COM receptors on the apical membranes of the TRG-treated cells [83]. TRG showed an anti-lithiatic effect in a nephrolithiatic rat model. In MDCK renal cells, TRG caused the upregulation of 23 proteins and the downregulation of 39 proteins. Functional enrichment and Reactome pathway analyses of these proteins suggested that TRG could prevent calcium oxalate monohydrate crystal-induced renal cell deterioration by inhibiting the crystal-induced overproduction of intracellular ROS, the G0/G1 to G2/M cell cycle shift, tight junction disruption, and EMT, providing evidence for the renoprotective effects of TRG on kidney stone formation [54].

## 8. Anti-Cancer Effects of TRG

TRG has been shown to have anti-cancer effects partially through the inhibition of the hyperactivation of Nrf2. Nrf2 is the master transcriptional factor for the maintenance of the cellular redox balance. Transient activation of Nrf2 defends against oxidative stress through cytoprotective and detoxifying activities. However, permanent activation or hyperactivation of Nrf2 is a detrimental factor, promoting cancer development, malignant progression, chemo/radioresistance, and poor patient prognosis [84,85,86]. Viruses and cancer cells can hijack the Nrf2 pathway to sustain the survival of virally infected cells to avoid a large increase in ROS in the presence of such pathologies [86]. Nrf2 also cross-talks with oncogenic pathways including those involving heat shock factor1 (HSF1), mammalian target of rapamycin (mTOR), and mutant (mut) p53 [86]. The interplay between mutp53 and Nrf2 can increase the survival of cancer cells [87,88]. The mutp53/HSP90 interaction activates a feedback loop between Nrf2 and p62 that induces chemoresistance in both pancreatic and breast cancer cells [89]. The interactions among mutp53, Nrf2, and HIF-1 can sustain their oncogenic functions and promote tumor progression, invasion, and chemoresistance [86]. Therefore, the inhibition of Nrf2 activity, such as by TRG, may be an effective approach for sensitizing cancer cells to anti-cancer therapy [84,85,86].

### 8.1. Anti-Head-and-Neck Cancer (HNC) Effects (Figure 1G)

Ferroptotic resistance is related to the upregulation of Nrf2. The activation of Nrf2 could cause chemo-sensitive HN3 cells to be resistant to RSL3. TRG was shown to sensitize RSL3 chemoresistant HN3R cells in an animal model. Thus, ferroptotic resistance in HNC caused by an increase in Nrf2–ARE pathway activation could be reversed by the blockage of this pathway [90]. Artesunate selectively killed HNC cells but not normal ones by a ferroptosis-dependent mechanism. However, in many cisplatin-resistant HNCs, in which the Nrf2–ARE pathway remained active, such an effect of artesunate was suboptimal. TRG could inhibit the Nrf2–ARE pathway, enhance ferroptosis, and sensitize artesunate-resistant HNC cells [91]. The inhibition of the Nrf2–ARE pathway by TRG plus the dual suppression of the GSH and thioredoxin (Trx) antioxidant systems could enhance the elimination of resistant HNC cells [92].

### 8.2. Anti-Lung Cancer and -Colon Cancer Effects (Figure 1G)

TRG inhibited Nrf2 activation and nuclear translocation in non-small cell lung cancer (NSCLC), inhibiting the EGFR signaling pathway and its downstream effector ERK 1/2 kinase [58]. TRG induced lung cancer cell cycle arrest and apoptosis and restored Nrf2, NF-κB p65, Bcl-2, cyclin D1, ICAM-1, and MMP-2 expression, along with increasing the levels of cGMP and active caspase-3 [48]. TRG-loaded micelles as potent Nrf2 inhibitors could be considered a promising tool to overcome oxaliplatin resistance in colon cancer patients [49]. TRG was shown to block the Nrf2-dependent expression of proteasomal genes (*s5a*/*psmd4* and *α5*/*psma5*) and reduce proteasome activity in pancreatic carcinoma cell lines (Panc1, Colo357, and MiaPaca2) and H6c7 pancreatic duct cells, leading to apoptosis in these cells [50].

### 8.3. Anti-Cancer Cell Migration and Anti-Lipoblastoma Effects (Figure 1G)

TRG-incorporated chitosan nanoparticles effectively inhibited tumor cell invasion [93]. TRG inhibited hepatoma cell migration by downregulating the Raf/ERK/Nrf2 signaling pathway and decreasing the expression of matrix metalloproteinases 7 (MMP-7) [94]. TRG-loaded water-soluble chitosan nanoparticles (Trigo-WSCS NPs) showed their anti-cancer efficacy in lipoblastoma (C6 glioma cells) [95].

## 9. Antiviral, Antimicrobial, and Antifungal Effects of TRG

### 9.1. Antiviral Effects (Figure 1H)

TRG showed antiviral effects against respiratory syncytial virus (RSV), the DNA virus herpes simplex type 1 (HSV-1), the RNA virus parainfluenza (type-3) (PI-3), wild-type Rift Valley fever virus (RVFV), Epstein–Barr virus (EBV), human gammaherpesvirus, and SARS-CoV-2-induced pathologies involving the spike protein. Toll-like receptor 7 (TLR7) upregulation was related to RSV infection and subsequent oxidative stress induction. TRG suppressed the RSV infection-mediated upregulation of ˙OH, NO, TLR7, IL-6, TNF-α/β, and IL-1β [96]. As regards the effects against the DNA virus herpes simplex type 1 (HSV-1) and the RNA virus parainfluenza (type-3) (PI-3), the cytopathic effect (CPE) inhibitory concentration of TRG for HSV-1 in MDBK cells and PI-3 in Vero cells was 1.6 µg/mL [97].

TRG prevented spike protein-exaggerated lipotoxicity, which might lead to cell death [98]. In addition, chlorogenic acid, ferulic acid, vanillic acid, and TRG were identified as the most prominent substances in 20 medicinal plants exerting biological activities against wild-type Rift Valley fever virus (RVFV), using NMR spectroscopy [99]. EBV can activate and stabilize Nrf2 in monocytic cells and impair the in vitro differentiation of monocytes into dendritic cells favoring immune escape, which may indirectly promote cancer onset [100]. The human gammaherpesvirus-mediated activation of Nrf2 increases viral replication and cell survival/proliferation of virally infected cells, thus contributing to carcinogenesis [101,102]. TRG is posited to attenuate the pathological effects caused by these viruses through the inhibition of Nrf2.

### 9.2. Antimicrobial Effects (Figure 1H)

The minimum inhibitory concentrations (MICs) of TRG ranged from 4 to 8 µg/mL for the ATCC strains *Acinetobacter baumannii*, *Bacillus subtilis*, *Escherichia coli*, *Enterococcus faecalis*, *Klebsiella pneumoniae*, *Proteus mirabilis*, *Pseudomonas aeruginosa*, and *Staphylococcus aureus* and from 32 to 64 µg/mL for their corresponding isolated ESµL+ strains. As a reference, the MICs of ampicillin ranged from 0.5 to 2 µg/mL for those ATCC strains and were >128 µg/mL for the isolated ESµL+ strains, respectively [97].

### 9.3. Antifungal and Antiparasitic Effects (Figure 1H)

The MICs of TRG for *Candida albicans* and *Candida parapsilosis* were 4 and 8 µg/mL, respectively. As a reference, the MICs of fluconazole for *C. albicans* and *C. parapsilosis* were 2 and 4 µg/mL, respectively [97]. TRG showed an impact on parasites such as *Echinococcus granulosus* which caused cystic echinococcosis or hydatid disease in various organs in humans. TRG could induce damage to *Echinococcus granulosus* protoscoleces, including hook deformation, lesions, and digitiform protuberances, by decreasing Nrf2, NQO-1, and HO-1 expression and increasing caspase-3 activity [103].

## 10. Other Protective Effects of TRG

### 10.1. Skin Protection Effects (Figure 1I)

TRG exhibited anti-melanogenic effects by inhibiting tyrosinase via competitive binding to its active site. The IC50 value of TRG for tyrosinase inhibition was about ~3.2 µM, stronger than kojic acid. A dose of 5 µM TRG was shown to inhibit 75% of tyrosinase activity in vitro [55] (Figure 2I). TRG could reduce UV-B-induced skin photodamage. TRG prevented UV-B-induced cell apoptosis in human skin fibroblasts via reducing oxidative stress, restoring Ca2+ homeostasis, re-establishing the ER function, and preventing the apoptotic process. Topical TRG protected mice skin from UV-B-induced apoptosis [45]. TRG prevented UV-B-induced oxidative stress by activating the PI3K/Akt/Nrf2 signaling pathway [46]. TRG prevented UV-B-induced collagen degradation and lipid peroxidation in Hs68 cells by attenuating MMP1 activity and prevented UV-B-induced cellular oxidative damage by modulating the ROS/MAPK/NF-κB axis [47].

### 10.2. Anti-Allergic Inflammation Effect

TRG was shown to suppress mast cell activation, alleviate pathological damage in lung tissue, and reduce the levels of serum immunoglobulin E (IgE) and T helper 2 cytokines [104]. TRG could mitigate the intracellular calcium-dependent and independent pathways and suppress the degranulation of IgE-sensitized mast cells, thus exerting an anti-degranulation effect against the development of allergic conditions [105].

### 10.3. Gastroprotective Effects (Figure 1J)

In an indomethacin-induced gastric ulcer rat model, TRG (45 mg/kg) pretreatment could inhibit lesion formation by 81.71% through increasing the levels of PGE2 and antioxidants (such as SOD, CAT, and GPx) and reducing the levels of pro-inflammatory molecules such as leukotriene B4 (LTB4), IL-6, IL-1β, TNF-α, interferon-γ (IFN-γ), IL-10, and IL-4. The protective activity of TRG against indomethacin-induced gastric ulcers was associated with its effects on anti-inflammatory, antioxidant, and anti-apoptotic pathways [43]. TRG effectively mitigated colitis and cardiomyopathy in a dextran sodium sulfate (DSS)-induced inflammatory bowel disease (IBD) mouse model. It could reduce the levels of MDA, TNF-α, IL-1β, and TLR4, improve histopathological alterations in the intestine, increase the antioxidant capacity, and attenuate the cardiac manifestations of colitis [44].

### 10.4. Phytoestrogenic Effects (Figure 1K)

TRG could increase the levels of testosterone. In a study on testosterone deficiency syndrome (TDS) treatment, a TRG-enriched extract of TFGL (*Trigonella foenum-graecum* seed and lespedeza cuneata) improved the AMS scores, ADAM scores, total testosterone levels, and free testosterone levels. The TRG-enriched extract also improved the levels of TC, HDL-c, LDL-c, TG, and the scores of IIEF and PSS-10 [106].

TRG could act as a phytoestrogen. TRG was shown to stimulate MCF-7 cell proliferation in a dose-responsive manner at concentrations as low as 100 pmol/L, and this effect could be blocked by an estrogen receptor (ER) antagonist. TRG increased the expression of ER target genes. TRG did not compete with E2 for ER, suggesting that TRG activated ER through a separate mechanism [52] (Figure 2D). TRG blocked the cell cycle in the G1/S phase by reducing c-Myc expression and increasing caspase-3 activity, thus inducing apoptosis in young adult mouse colonocytes (YAMCs). These effects of TRG could be blocked by ICI 182,780, an ER antagonist, suggesting that TRG impact in the colon might be depended on novel estrogenic actions [107].

### 10.5. Bone Density Regulation (Figure 1L)

TRG was shown to exert an estrogenic activity, preventing the dexamethasone-induced progression of osteoporosis by enhancing bone mineral density (BMD) and restoring bone physiology [108]. TRG increased bone density in non-hyperglycemic rats. In STZ-induced diabetic rats, TRG decreased bone mineralization and tended to worsen the bone mechanical properties. In nicotinamide/STZ-treated rats (with only a slight increase in blood glucose level), TRG increased BMD and tended to improve cancellous bone strength. TRG appeared to affect the skeletal system of rats with STZ-induced metabolic disorders, intensifying osteoporotic changes, and favorably affecting bones in non-hyperglycemic (nicotinamide/STZ-treated) rats [109].

### 10.6. Extending the Lifespan (Figure 1M)

TRG (50 μM) could prolong about 17.9% of lifespan of Caenorhabditis elegans (*C. elegans*), showing an anti-aging effect [110]. Under physiological conditions, the Keap1–Nrf2 pathway defends cells from oxidation and electrophiles. However, Keap1 knockout is lethal in zebrafish, due to the hyperactivation of Nrf2, which increases the expression of Nrf2-target genes and decreases the expression of visual cycle genes. TRG could partially rescue Keap1-knockout larvae from death [111].

## 11. Discrepancy Regarding TRG Effects in the Literature

As an inhibitor of Nrf2, TRG was shown to attenuate the effects that were exerted by some Nrf2 activating compounds. For example, plumbagin (5-hydroxy-2-methyl-1,4-naphthoquinone) could protect against cerebral ischemia- and spinal cord injury-induced oxidative stress and inflammation by activating the Nrf2–ARE pathway in an STZ-induced AD-like mouse model, which could be ameliorated by TRG [112]. Shinorine is a mycosporine-like amino acid and a mammalian Keap1 antagonist. It was shown to ameliorate the hepatotoxic effects induced by chromium, a heavy metal toxicant, by acting as a disruptor of Nrf2–Keap1 interaction. TRG could neutralize the alleviative effect of shinorine [113]. Nitro-oleic acid showed beneficial effects against oxidative stress, gliosis, and the pro-angiogenic response in Müller glial cells through the activation of the Nrf2 pathway in individuals with proliferative retinopathies; such an action could be abrogated by TRG [114]. Ang (1–7) exhibited Nrf2-mediated antioxidant activity against Aβ-induced mitochondrial dysfunction and neurotoxicity in an AD male rat model; such an effect could be ameliorated by TRG [115]. TRG was shown to abolish the analgesic effect of rosiglitazone against paclitaxel-induced neuropathic pain through the inhibition of the Nrf2 pathway [116]. Berberine could alleviate doxorubicin-induced cardiotoxicity by activating the Nrf2-mediated pathway; such an effect could be attenuated by TRG [117]. Astaxanthin appeared to act as a radical scavenger and an anti-apoptotic factor through the activation of the Nrf2 pathway in cultured human primary granulosa cells; such an effect could be attenuated by TRG [118]. The neuroprotective effect of dimethyl fumarate in a mouse model of PD was mediated by the activation of Nrf2, which could be blocked by TRG [119].

The precise reasons underlying the TRG-induced dichotomous effects remain unclear. We have demonstrated that Nrf2 is required for cardiac adaptation when cardiac autophagy is intact; however, Nrf2 operates a pathological program to exacerbate maladaptive cardiac remodeling and dysfunction when myocardial autophagy is inhibited in the settings of sustained pressure overload [120] and chronic type 1 diabetes [121]. We proposed that normal autophagy is required for Nrf2-mediated cardiac protection, whereas autophagy impairment switches on an Nrf2-operated pathological program leading to myocardial damage and dysfunction [122]. Extrapolating this concept to other organ systems, it is likely that during acute exposure to insults or stresses, autophagy may be intact, and thus Nrf2 activation is protective; once the insults or stresses are persistent, autophagy may be impaired, thereby turning on Nrf2-operated detrimental signaling, while Nrf2-mediated cytoprotection is exhausted. For example, it was demonstrated that Nrf2 activation was detrimental in the autophagy-impaired liver [123]. In addition, like Nrf2-mediated ferroptosis in type 1 diabetic heart [121], autophagy impairment was likely to turn on Nrf2-mediated renal injury induced by chronic iron exposure [82]. Therefore, except for the potential non-Nrf2-targeted effects of TRG, the observed dual effects of TRG are most likely linked to the Nrf2-operated dichotomous actions.

## 12. Conclusions

In summary, TRG functions as an anti-inflammation and antioxidation agent, showing various beneficial effects on many organs and tissues. It can (1) exert a metabolic modulation of glucose and lipids, (2) help recover from nervous system abnormalities such as neurodegenerative disorders, ischemia-induced brain damage, depression, cognitive impairments, and diabetic peripheral neuropathy, (3) mitigate conditions related to DM and its complications, (4) protect the cardiovascular system, liver, lungs, kidney, gastric system, and skin, and (5) suppress tumor cell proliferation and migration (Figure 1, Table 1). It exhibits great potential as a natural, systematic health booster, with a good safety profile.

There are several limitations in our understanding of TRG functions, which will require future studies. (1) The mechanism remains unclear of how TRG penetrates the blood–brain barrier, though evidence shows that TRG can be detected in the cortex after oral administration [16]. It will be essential to understand its cerebral pharmacokinetics to gain insight into its therapeutic potential in the nervous system. (2) Molecular docking data showed that TRG can bind to multiple targets, such as PPARγ, GSK, tyrosinase, NGF, Aβ, ER, and several neurotransmitter receptors (Figure 2). However, relevant biological function data is very limited. For example, two studies suggested that TRG might bind to the PPARγ, thus regulating its activity [5,30]; however, TRG induced the suppression of PPARγ expression in 3T3-L1 cells [5]. Other studies showed that TRG enhanced PPARγ expression in the adipose tissue of T2DM mice [30] and T2DM rats [13]. This discrepancy is probably due to different experimental models, e.g., insulin-sensitive versus insulin-resistant adipocytes. Therefore, further characterization of their intermolecular actions and altered molecular activities is needed to address the mechanisms underlying these various outcomes. (3) As an Nrf2 inhibitor, TRG may exert dichotomous functions that depend on whether Nrf2 exerts a protective or a detrimental role in a particular pathological status. The current data support the hypothesis that Nrf2 over-activation or exhaustion under chronic conditions or tumorigenesis will accelerate the pathological progression; thus, TRG might exert a beneficial effect on these conditions. However, this needs more investigations to understand the underlying mechanisms. (4) More clinical trials are needed to validate these data, particularly the TRG effects on neuroprotection and DM and its complications.

## Figures and Tables

**Table 1:** A summary of the pharmacological activities of TRG, experimental models, potential mechanisms, and natural sources.

Organs/Pathological Conditions	Pharmacological Effects	Experimental Models	Potential Signaling Pathways/Targets	Compound/Natural Sources	Refs
Aging	Mitochondria protection (Section 10.6); increasing lifespan (Section 10.6)	*C. elegans*; Zebrafish	AMPK; DAF-16; Suppressing hyperactivation of Nrf2	TRG	[110,111]
Cardiovascular system	Decreasing cardiomyopathy (Section 6.1)	H9C2 cells; isolated gut microbe; DSS-induced IBD mouse model	Anti-apoptotic pathway; FMOs	TRG; TRG extracted from *Trigonella foenum-graecum* seeds	[44,76,78]
	Decreasing myocardial injury (Section 6.2)	NICO/STZ-induced DM rats; ISO-induced rats	Downregulation of Hsp27, alphaB-crystallin, and CaMKII delta	TRG; TRG isolated from fenugreek seeds	[33,34]
	Decreasing fibrosis/inhibiting EMT (Section 6.3)	In vitro turbidity assay; BLM-induced pulmonary fibrosis	Inhibiting NF-κB/NLRP3/IL-1β	TRG	[53,79]
	Improving endothelial cell function (Section 6.4)	Human	n.a.	TRG-enriched Sakurajima radish	[80]
Inflammation	Anti-inflammatory effects (Section 5.2 and Section 6.3)	BLM-induced pulmonary fibrosis; HFHF IR rats	Inhibiting NF-κB/NLRP3/IL-1β	TRG	[35,53]
	Anti-allergic effects (Section 10.2)	RBL-2H3 cells; PCA reaction, mice; OVA-induced asthma model	Inhibiting intracellular calcium-dependent and -independent pathways; HIF-1alpha	TRG	[104,105]
Kidney	Decreasing DM nephropathy (Section 7.1)	Oxalate-induced EMT; db/db DKD mice; HMCs; neonatal diabetic rats; STZ-induced T2DM rats	Anti-EMT pathway; inhibiting TNF-α signaling; anti-Wnt/b-catenin signaling; AMPK; Smad7	TRG	[37,38,39,40,41,42]
	Decreasing metal-induced kidney injury (Section 7.2)	PTCs	Inhibiting hyperactivated Nrf2 signaling	TRG	[82]
	Decreasing stone formation (Section 7.3)	MDCK renal tubular cells	n.a.	TRG	[54,83]
Liver	Decreasing steatosis (Section 5.1)	HC-HFD mice	Modulating autophagy	TRG	[74]
	Decreasing NAFLD injury (Section 5.1)	HFD rats	Anti-apoptotic pathway	TRG	[75]
	Improving liver function (Section 5.2)	HFHF IR rats; HepG2 cells	Anti-inflammatory and antioxidative pathways	TRG	[35,36]
Glucose and lipid metabolism	Decreasing glucose synthesis and transport (Section 2.1); hypoglycemic effects (Section 3.3)	T2DM-GK rats; overweight men; T2DM KK-A^y^ obese mouse; molecular docking simulation; HFD mice	GSK-3a; GSK-3b	TRG; GTF-231 (gymnemic acid, TRG, and ferulic acid in the ratio of 2:3:1); TRG-enriched yogurt	[4,5,6,7,8,9,10]
	Decreasing lipogenesis and fatty acid levels (Section 2.2)	T2DM-GK rats; 3T3-L1 cells	PPARγ; p38/ATF-2; inhibiting TNF-α signaling	TRG	[4,5,63,64]
Nervous system	Peripheral neuropathy (Section 4.1)	STZ HCHF T2DM rats; LepR(db/db) mice; docking simulation; alloxan-induced diabetic zebrafish	p38 MAPK; NGF	TRG	[24,25]
	Neuronal protection in AD and PD (Section 4.2 and Section 4.3)	Aβ-induced AD rat model; 5XFAD mouse model; HILIC; 6-OHDA-induced PD rats;	Aβ; CKB	TRG	[15,16,17,18]
	Cognitive improvement (Section 4.4)	LPS-induced cognitive dysfunction; D-gal-induced amnesia model; SAMP8 mice	TLR4/NF-kB; Traf6-NF-kB	TRG	[21,22,23]
	Decreasing stroke-induced brain injury (Section 4.5)	MCAo ischemic stroke rat model; OGD/R mouse model	MPO; PI3K/Akt	TRG	[19,20]
	Anti-depression and anti-epilepsy (Section 4.6)	FST-induced mice; MS stress-induced depressive- and anxiety-like mouse model; kainic acid-induced epileptic model	Anti-inflammatory and antioxidative pathways	TRG	[26,27,28]
	Neuromodulation effects (Section 4.7)	Molecular docking simulation	GABARS, mAChR, 5HTRs, NMDAR, AMPAR	TRG	[51]
Oxidation	Antioxidative stress (Section 3.2)	Alloxan diabetic rabbits; STZ HCHF T2DM rats; STZ–HFD rats	PPARγ; inhibiting TNF-α signaling; increasing SOD, CAT, GSH	TRG; Iraqi fenugreek seed extracts; *Trigonella stellata*	[11,12,13,14]
Pancreas	Increasing insulin sensitivity (Section 3.4)	STZ HFD T2DM rats; DM patients	Insulin receptor	TRG; fenugreek seed	[68,69,70]
	Protecting β-cells and improving β-cell function (Section 3.1 and Section 3.2)	STZ-induced DM mice; T2DM rats; alloxan-induced diabetic rats; diabetic pregnant mice	PPARγ; anti-apoptotic pathway	TRG	[6,11,29,30,31,32]
Pathogen infections	Anti-viral effects (Section 9.1)	RSV, HSV-1, PI-3, RVFV, EBV, human gammaherpesvirus, spike protein of SARS-CoV-2	Inhibiting TLR7 signaling; inhibiting hyperactivated Nrf2 signaling	TRG	[96,97,98,99,100,101,102]
	Anti-bacterial effects (Section 9.2)	*A. baumannii*, *B. subtilis*, *E. coli*, *E. faecalis*, *K. pneumoniae*, *P. mirabilis*, *P. aeruginosa*, and *S. aureus*	n.a.	TRG	[97]
	Antifungal effects (Section 9.3)	*C. albicans* and *C. parapsilosis*	n.a.	TRG	[97]
	Antiparasitic effects (Section 9.3)	*Echinococcus granulosus*	Inhibiting hyperactivated Nrf2 signaling	TRG	[103]
Skin	Anti-melanogenic effects (Section 10.1)	Molecular docking simulation; in vitro kinetic assay	Inhibiting tyrosinase	Emulgels containing fenugreek extract and fenugreek extract-entrapped niosomes	[55]
	Decreasing UVB-induced photoaging (Section 10.1)	human skin fibroblasts; UVB-exposed mouse skin; Hs68 cells;	Inhibiting ROS/MAPK/NF-kB	TRG	[45,46,47]
Tumor	Inhibition of tumor cell proliferation/increasing chemo-sensitivity (Section 8.1, Section 8.2 and Section 8.3)	HNC, NSCLC, colon cancer cells	Inhibiting hyperactivated Nrf2 signaling	TRG, TRG-loaded micelles	[48,49,50]
	Inhibition of tumor cell migration (Section 8.3)	Hepatoma cell	Inhibiting Raf/ERK/Nrf2	TRG-loaded water-soluble chitosan nanoparticles	[93,94]
Others	Bone density regulation (Section 10.5)	Nicotinamide/STZ rats; STZ rats; dexamethasone-induced osteoporosis;	n.a.	TRG	[108,109]
	Phytoestrogen effects (Section 10.4)	Human subjects with TDS; MCF-7 cells; YAMCs	ER	TRG-enriched extract of TFGL (*Trigonella foenum-graecum* seed and lespedeza cuneata); TRG	[52,106,107]
	Mitigation of gastric ulcer and IBD (Section 10.3)	Indomethacin-induced gastric ulcer rat model; DSS-induced IBD mouse model	anti-inflammatory, antioxidant, and anti-apoptotic pathways	TRG	[43,44]

n.a., no data available.

## References

[1] Johns E. (1885). Ueber die alkaloide des bockshornsamens. Berdeutchem. Ges..

[2] Taguchi H., Sakaguchi M., Shimabayashi Y. (1985). Trigonelline content in coffee beans and the thermal conversion of trigonelline into nicotinic acid during the roasting of coffee beans. Agric. Biol. Chem..

[3] Ashihara H. (2015). Chapter 3—Plant biochemistry: Trigonelline biosynthesis in coffea arabica and coffea canephora. Coffee in Health and Disease Prevention.

[4] Yoshinari O., Takenake A., Igarashi K. (2013). Trigonelline ameliorates oxidative stress in type 2 diabetic goto-kakizaki rats. J. Med. Food.

[5] Ilavenil S., Arasu M.V., Lee J.C., Kim D.H., Roh S.G., Park H.S., Choi G.J., Mayakrishnan V., Choi K.C. (2014). Trigonelline attenuates the adipocyte differentiation and lipid accumulation in 3t3-l1 cells. Phytomedicine.

[6] Hamden K., Mnafgui K., Amri Z., Aloulou A., Elfeki A. (2013). Inhibition of key digestive enzymes related to diabetes and hyperlipidemia and protection of liver-kidney functions by trigonelline in diabetic rats. Sci. Pharm..

[7] Devi V.R., Sharmila C., Subramanian S. (2018). Molecular docking studies involving the inhibitory effect of gymnemic acid, trigonelline and ferulic acid, the phytochemicals with antidiabetic properties, on glycogen synthase kinase 3 (α and β). J. Appl. Pharm. Sci..

[8] Vellai R.D., Chandiran S., Pillai S.S. (2018). Gtf-231, a mixture of gymnemic acid, trigonelline and ferulic acid significantly ameliorates oxidative stress in experimental type 2 diabetes in rats. Can. J. Diabetes.

[9] Yoshinari O., Igarashi K. (2010). Anti-diabetic effect of trigonelline and nicotinic acid, on kk-a(y) mice. Curr. Med. Chem..

[10] Costa M.C., Lima T.F.O., Arcaro C.A., Inacio M.D., Batista-Duharte A., Carlos I.Z., Spolidorio L.C., Assis R.P., Brunetti I.L., Baviera A.M. (2020). Trigonelline and curcumin alone, but not in combination, counteract oxidative stress and inflammation and increase glycation product detoxification in the liver and kidney of mice with high-fat diet-induced obesity. J. Nutr. Biochem..

[11] Zhou J., Zhou S., Zeng S. (2013). Experimental diabetes treated with trigonelline: Effect on beta cell and pancreatic oxidative parameters. Fundam. Clin. Pharmacol..

[12] Hamadi S.A. (2012). Effect of trigonelline and ethanol extract of iraqi fenugreek seeds on oxidative stress in alloxan diabetic rabbits. J. Assoc. Arab Univ. Basic Appl. Sci..

[13] Li Y., Li Q., Wang C., Lou Z., Li Q. (2019). Trigonelline reduced diabetic nephropathy and insulin resistance in type 2 diabetic rats through peroxisome proliferator-activated receptor-gamma. Exp. Ther. Med..

[14] Sheweita S.A., ElHady S.A., Hammoda H.M. (2020). *Trigonella stellata* reduced the deleterious effects of diabetes mellitus through alleviation of oxidative stress, antioxidant- and drug-metabolizing enzymes activities. J. Ethnopharmacol..

[15] Makowska J., Szczesny D., Lichucka A., Gieldon A., Chmurzynski L., Kaliszan R. (2014). Preliminary studies on trigonelline as potential anti-alzheimer disease agent: Determination by hydrophilic interaction liquid chromatography and modeling of interactions with beta-amyloid. J. Chromatogr. B Anal. Technol. Biomed. Life Sci..

[16] Farid M.M., Yang X., Kuboyama T., Tohda C. (2020). Trigonelline recovers memory function in alzheimer’s disease model mice: Evidence of brain penetration and target molecule. Sci. Rep..

[17] Fahanik-Babaei J., Baluchnejadmojarad T., Nikbakht F., Roghani M. (2019). Trigonelline protects hippocampus against intracerebral aβ(1–40) as a model of alzheimer’s disease in the rat: Insights into underlying mechanisms. Metab. Brain Dis..

[18] Mirzaie M., Khalili M., Kiasalari Z., Roghani M. (2016). Neuroprotective and antiapoptotic potential of trigonelline in a striatal 6-hydroxydopamine rat model of parkinson’s disease. Neurophysiology.

[19] Pravalika K., Sarmah D., Kaur H., Vats K., Saraf J., Wanve M., Kalia K., Borah A., Yavagal D.R., Dave K.R. (2019). Trigonelline therapy confers neuroprotection by reduced glutathione mediated myeloperoxidase expression in animal model of ischemic stroke. Life Sci..

[20] Qiu Z., Wang K., Jiang C., Su Y., Fan X., Li J., Xue S., Yao L. (2020). Trigonelline protects hippocampal neurons from oxygen-glucose deprivation-induced injury through activating the pi3k/akt pathway. Chem. Biol. Interact..

[21] Chowdhury A.A., Gawali N.B., Munshi R., Juvekar A.R. (2018). Trigonelline insulates against oxidative stress, proinflammatory cytokines and restores bdnf levels in lipopolysaccharide induced cognitive impairment in adult mice. Metab. Brain Dis..

[22] Chowdhury A.A., Gawali N.B., Bulani V.D., Kothavade P.S., Mestry S.N., Deshpande P.S., Juvekar A.R. (2018). In vitro antiglycating effect and in vivo neuroprotective activity of trigonelline in d-galactose induced cognitive impairment. Pharmacol. Rep..

[23] Aktar S., Ferdousi F., Kondo S., Kagawa T., Isoda H. (2023). Transcriptomics and biochemical evidence of trigonelline ameliorating learning and memory decline in the senescence-accelerated mouse prone 8 (samp8) model by suppressing proinflammatory cytokines and elevating neurotransmitter release. Geroscience.

[24] Zhou J.Y., Zhou S.W. (2012). Protection of trigonelline on experimental diabetic peripheral neuropathy. Evid. Based Complement. Altern. Med..

[25] Castaneda R., Rodriguez I., Nam Y.H., Hong B.N., Kang T.H. (2017). Trigonelline promotes auditory function through nerve growth factor signaling on diabetic animal models. Phytomedicine.

[26] Anjomshoa M., Boroujeni S.N., Bagheri E., Lorigooini Z., Amini-Khoei H. (2020). Possible involvement of n-methyl-d-aspartate receptor (nmda-r) in the antidepressant- like effect of trigonelline in male mice. Curr. Pharm. Des..

[27] Lorigooini Z., Sadeghi Dehsahraei K., Bijad E., Habibian Dehkordi S., Amini-Khoei H. (2020). Trigonelline through the attenuation of oxidative stress exerts antidepressant- and anxiolytic-like effects in a mouse model of maternal separation stress. Pharmacology.

[28] Faizan M., Jahan I., Ishaq M., Alhalmi A., Khan R., Noman O.M., Hasson S., Mothana R.A. (2023). Neuroprotective effects of trigonelline in kainic acid-induced epilepsy: Behavioral, biochemical, and functional insights. Saudi Pharm. J..

[29] Liu L., Du X., Zhang Z., Zhou J. (2018). Trigonelline inhibits caspase 3 to protect beta cells apoptosis in streptozotocin-induced type 1 diabetic mice. Eur. J. Pharmacol..

[30] Tharaheswari M., Jayachandra Reddy N., Kumar R., Varshney K.C., Kannan M., Sudha Rani S. (2014). Trigonelline and diosgenin attenuate er stress, oxidative stress-mediated damage in pancreas and enhance adipose tissue ppargamma activity in type 2 diabetic rats. Mol. Cell. Biochem..

[31] Hamden K., Bengara A., Amri Z., Elfeki A. (2013). Experimental diabetes treated with trigonelline: Effect on key enzymes related to diabetes and hypertension, beta-cell and liver function. Mol. Cell. Biochem..

[32] Zhou J.Y., Du X.H., Zhang Z., Qian G.S. (2017). Trigonelline inhibits inflammation and protects beta cells to prevent fetal growth restriction during pregnancy in a mouse model of diabetes. Pharmacology.

[33] Kamble H.V., Bodhankar S.L. (2014). Cardioprotective effect of concomitant administration of trigonelline and sitagliptin on cardiac biomarkers, lipid levels, electrocardiographic and heamodynamic modulation on cardiomyopathy in diabetic wistar rats. Biomed. Aging Pathol..

[34] Panda S., Biswas S., Kar A. (2013). Trigonelline isolated from fenugreek seed protects against isoproterenol-induced myocardial injury through down-regulation of hsp27 and alphab-crystallin. Nutrition.

[35] Afifi N.A., Ramadan A., Erian E.Y., Saleh D.O., Sedik A.A., Badawi M., El Hotaby W. (2017). Trigonelline attenuates hepatic complications and molecular alterations in high-fat high-fructose diet-induced insulin resistance in rats. Can. J. Physiol. Pharmacol..

[36] Peerapen P., Chanthick C., Thongboonkerd V. (2023). Quantitative proteomics reveals common and unique molecular mechanisms underlying beneficial effects of caffeine and trigonelline on human hepatocytes. Biomed. Pharmacother..

[37] Peerapen P., Thongboonkerd V. (2020). Protective roles of trigonelline against oxalate-induced epithelial-to-mesenchymal transition in renal tubular epithelial cells: An in vitro study. Food Chem. Toxicol..

[38] Gong M., Guo Y., Dong H., Wu W., Wu F., Lu F. (2023). Trigonelline inhibits tubular epithelial-mesenchymal transformation in diabetic kidney disease via targeting smad7. Biomed. Pharmacother..

[39] Chen C., Ma J., Miao C.S., Zhang H., Zhang M., Cao X., Shi Y. (2021). Trigonelline induces autophagy to protect mesangial cells in response to high glucose via activating the mir-5189-5p-ampk pathway. Phytomedicine.

[40] Ghule A.E., Jadhav S.S., Bodhankar S.L. (2012). Trigonelline ameliorates diabetic hypertensive nephropathy by suppression of oxidative stress in kidney and reduction in renal cell apoptosis and fibrosis in streptozotocin induced neonatal diabetic (nstz) rats. Int. Immunopharmacol..

[41] Shao X., Chen C., Miao C., Yu X., Li X., Geng J., Fan D., Lin X., Chen Z., Shi Y. (2019). Expression analysis of micrornas and their target genes during experimental diabetic renal lesions in rats administered with ginsenoside rb1 and trigonelline. Pharmazie.

[42] Zhao S., Ghosh A., Lo C.S., Chenier I., Scholey J.W., Filep J.G., Ingelfinger J.R., Zhang S.L., Chan J.S.D. (2018). Nrf2 deficiency upregulates intrarenal angiotensin-converting enzyme-2 and angiotensin 1–7 receptor expression and attenuates hypertension and nephropathy in diabetic mice. Endocrinology.

[43] Antonisamy P., Arasu M.V., Dhanasekaran M., Choi K.C., Aravinthan A., Kim N.S., Kang C.W., Kim J.H. (2016). Protective effects of trigonelline against indomethacin-induced gastric ulcer in rats and potential underlying mechanisms. Food Funct..

[44] Omidi-Ardali H., Lorigooini Z., Soltani A., Balali-Dehkordi S., Amini-Khoei H. (2019). Inflammatory responses bridge comorbid cardiac disorder in experimental model of ibd induced by dss: Protective effect of the trigonelline. Inflammopharmacology.

[45] Nazir L.A., Tanveer M.A., Shahid N.H., Sharma R.R., Tasduq S.A. (2020). Trigonelline, a naturally occurring alkaloidal agent protects ultraviolet-b (uv-b) irradiation induced apoptotic cell death in human skin fibroblasts via attenuation of oxidative stress, restoration of cellular calcium homeostasis and prevention of endoplasmic reticulum (er) stress. J. Photochem. Photobiol. B.

[46] Tanveer M.A., Rashid H., Nazir L.A., Archoo S., Shahid N.H., Ragni G., Umar S.A., Tasduq S.A. (2023). Trigonelline, a plant derived alkaloid prevents ultraviolet-b-induced oxidative DNA damage in primary human dermal fibroblasts and balb/c mice via modulation of phosphoinositide 3-kinase-akt-nrf2 signalling axis. Exp. Gerontol..

[47] Nazir L.A., Tanveer M.A., Umar S.A., Love S., Divya G., Tasduq S.A. (2021). Inhibition of ultraviolet-b radiation induced photodamage by trigonelline through modulation of mitogen activating protein kinases and nuclear factor-kappab signaling axis in skin. Photochem. Photobiol..

[48] Hamzawy M.A., Abo-Youssef A.M., Malak M.N., Khalaf M.M. (2022). Multiple targets of nrf 2 inhibitor; trigonelline in combating urethane-induced lung cancer by caspase-executioner apoptosis, cgmp and limitation of cyclin d1 and bcl2. Eur. Rev. Med. Pharmacol. Sci..

[49] Pirpour Tazehkand A., Salehi R., Velaei K., Samadi N. (2020). The potential impact of trigonelline loaded micelles on nrf2 suppression to overcome oxaliplatin resistance in colon cancer cells. Mol. Biol. Rep..

[50] Arlt A., Sebens S., Krebs S., Geismann C., Grossmann M., Kruse M.L., Schreiber S., Schafer H. (2013). Inhibition of the nrf2 transcription factor by the alkaloid trigonelline renders pancreatic cancer cells more susceptible to apoptosis through decreased proteasomal gene expression and proteasome activity. Oncogene.

[51] Zia S.R., Wasim M., Ahmad S. (2023). Unlocking therapeutic potential of trigonelline through molecular docking as a promising approach for treating diverse neurological disorders. Metab. Brain Dis..

[52] Allred K.F., Yackley K.M., Vanamala J., Allred C.D. (2009). Trigonelline is a novel phytoestrogen in coffee beans. J. Nutr..

[53] Zeyada M.S., Eraky S.M., El-Shishtawy M.M. (2023). Trigonelline mitigates bleomycin-induced pulmonary inflammation and fibrosis: Insight into nlrp3 inflammasome and sphk1/s1p/hippo signaling modulation. Life Sci..

[54] Peerapen P., Boonmark W., Putpeerawit P., Sassanarakkit S., Thongboonkerd V. (2023). Proteomic and computational analyses followed by functional validation of protective effects of trigonelline against calcium oxalate-induced renal cell deteriorations. Comput. Struct. Biotechnol. J..

[55] Masjedi M., Solhjoo A. (2022). Does trigonelline help skin tone? Molecular docking studies of trigonelline on the human tyrosinase, formulation, optimization, and characterization of an emulgel-containing *Trigonella foenum-graecum* L. Fenugreek standardized hydroalcoholic extract. J. Cosmet. Dermatol..

[56] Aswar U., Mohan V., Bodhankar S. (2009). Effect of trigonelline on fertility in female rats. Int. J. Green Pharm..

[57] Deshpande P., Mohan V., Reddy K., Manjunath V., Thakurdesai P. (2017). Prenatal developmental toxicity evaluation of idm01, a botanical composition of 4-hydroxyisoleucine and trigonelline based standardized fenugreek seed extract, during organogenesis period of pregnancy in rats. J. Appl. Pharm. Sci..

[58] Fouzder C., Mukhuty A., Mukherjee S., Malick C., Kundu R. (2021). Trigonelline inhibits nrf2 via egfr signalling pathway and augments efficacy of cisplatin and etoposide in nsclc cells. Toxicol. In Vitro.

[59] Konstantinidis N., Franke H., Schwarz S., Lachenmeier D.W. (2023). Risk assessment of trigonelline in coffee and coffee by-products. Molecules.

[60] Cheng Z.X., Wu J.J., Liu Z.Q., Lin N. (2013). Development of a hydrophilic interaction chromatography-uplc assay to determine trigonelline in rat plasma and its application in a pharmacokinetic study. Chin. J. Nat. Med..

[61] Mohamadi N., Sharififar F., Ansari M., Pournamdari M., Rezaei M., Hassanabadi N. (2021). Pharmacokinetic profile of diosgenin and trigonelline following intravenous and oral administration of fenugreek seed extract and pure compound in rabbit. J. Asian Nat. Prod. Res..

[62] van Dijk A.E., Olthof M.R., Meeuse J.C., Seebus E., Heine R.J., van Dam R.M. (2009). Acute effects of decaffeinated coffee and the major coffee components chlorogenic acid and trigonelline on glucose tolerance. Diabetes Care.

[63] Yoshinari O., Sato H., Igarashi K. (2009). Anti-diabetic effects of pumpkin and its components, trigonelline and nicotinic acid, on goto-kakizaki rats. Biosci. Biotechnol. Biochem..

[64] Choi M., Mukherjee S., Yun J.W. (2021). Trigonelline induces browning in 3t3-l1 white adipocytes. Phytother. Res..

[65] Yoshinari O., Igarashi K. (2015). Chapter 85—Antidiabetic effects of trigonelline: Comparison with nicotinic acid. Coffee in Health and Disease Prevention.

[66] MacAulay K., Doble B.W., Patel S., Hansotia T., Sinclair E.M., Drucker D.J., Nagy A., Woodgett J.R. (2007). Glycogen synthase kinase 3alpha-specific regulation of murine hepatic glycogen metabolism. Cell Metab..

[67] Friedrich N., Skaaby T., Pietzner M., Budde K., Thuesen B.H., Nauck M., Linneberg A. (2018). Identification of urine metabolites associated with 5-year changes in biomarkers of glucose homoeostasis. Diabetes Metab..

[68] Subramanian S.P., Prasath G.S. (2014). Antidiabetic and antidyslipidemic nature of trigonelline, a major alkaloid of fenugreek seeds studied in high-fat-fed and low-dose streptozotocin-induced experimental diabetic rats. Biomed. Prev. Nutr..

[69] Aldakinah A.A., Al-Shorbagy M.Y., Abdallah D.M., El-Abhar H.S. (2017). Trigonelline and vildagliptin antidiabetic effect: Improvement of insulin signalling pathway. J. Pharm. Pharmacol..

[70] Najdi R.A., Hagras M.M., Kamel F.O., Magadmi R.M. (2019). A randomized controlled clinical trial evaluating the effect of trigonella foenum-graecum (fenugreek) versus glibenclamide in patients with diabetes. Afr. Health Sci..

[71] Khalili M., Alavi M., Esmaeil-Jamaat E., Baluchnejadmojarad T., Roghani M. (2018). Trigonelline mitigates lipopolysaccharide-induced learning and memory impairment in the rat due to its anti-oxidative and anti-inflammatory effect. Int. Immunopharmacol..

[72] Pravalika K., Sarmah D., Kaur H., Wanve M., Saraf J., Kalia K., Borah A., Yavagal D.R., Dave K.R., Bhattacharya P. (2018). Myeloperoxidase and neurological disorder: A crosstalk. ACS Chem. Neurosci..

[73] Zhou J., Chan L., Zhou S. (2012). Trigonelline: A plant alkaloid with therapeutic potential for diabetes and central nervous system disease. Curr. Med. Chem..

[74] Sharma L., Lone N.A., Knott R.M., Hassan A., Abdullah T. (2018). Trigonelline prevents high cholesterol and high fat diet induced hepatic lipid accumulation and lipo-toxicity in c57bl/6j mice, via restoration of hepatic autophagy. Food Chem. Toxicol..

[75] Zhang D.F., Zhang F., Zhang J., Zhang R.M., Li R. (2015). Protection effect of trigonelline on liver of rats with non-alcoholic fatty liver diseases. Asian Pac. J. Trop. Med..

[76] Ilavenil S., Kim D.H., Jeong Y.I., Arasu M.V., Vijayakumar M., Prabhu P.N., Srigopalram S., Choi K.C. (2015). Trigonelline protects the cardiocyte from hydrogen peroxide induced apoptosis in h9c2 cells. Asian Pac. J. Trop. Med..

[77] Zeisel S.H., Warrier M. (2017). Trimethylamine n-oxide, the microbiome, and heart and kidney disease. Annu. Rev. Nutr..

[78] Anwar S., Bhandari U., Panda B.P., Dubey K., Khan W., Ahmad S. (2018). Trigonelline inhibits intestinal microbial metabolism of choline and its associated cardiovascular risk. J. Pharm. Biomed. Anal..

[79] Rasheeda K., Fathima N.N. (2018). Trigonelline hydrochloride: A promising inhibitor for type i collagen fibrillation. Colloids Surf. B Biointerfaces.

[80] Sasaki M., Nonoshita Y., Kajiya T., Atsuchi N., Kido M., Chu D.C., Juneja L.R., Minami Y., Kajiya K. (2020). Characteristic analysis of trigonelline contained in *Raphanus sativus* cv. *Sakurajima daikon* and results from the first trial examining its vasodilator properties in humans. Nutrients.

[81] Zhao S., Lo C.S., Miyata K.N., Ghosh A., Zhao X.P., Chenier I., Cailhier J.F., Ethier J., Lattouf J.B., Filep J.G. (2021). Overexpression of nrf2 in renal proximal tubular cells stimulates sodium-glucose cotransporter 2 expression and exacerbates dysglycemia and kidney injury in diabetic mice. Diabetes.

[82] van Raaij S.E.G., Masereeuw R., Swinkels D.W., van Swelm R.P.L. (2018). Inhibition of nrf2 alters cell stress induced by chronic iron exposure in human proximal tubular epithelial cells. Toxicol. Lett..

[83] Peerapen P., Boonmark W., Thongboonkerd V. (2022). Trigonelline prevents kidney stone formation processes by inhibiting calcium oxalate crystallization, growth and crystal-cell adhesion, and downregulating crystal receptors. Biomed. Pharmacother..

[84] Gjorgieva Ackova D., Maksimova V., Smilkov K., Buttari B., Arese M., Saso L. (2023). Alkaloids as natural nrf2 inhibitors: Chemoprevention and cytotoxic action in cancer. Pharmaceuticals.

[85] Pouremamali F., Pouremamali A., Dadashpour M., Soozangar N., Jeddi F. (2022). An update of nrf2 activators and inhibitors in cancer prevention/promotion. Cell Commun. Signal..

[86] Cirone M., D’Orazi G. (2022). Nrf2 in cancer: Cross-talk with oncogenic pathways and involvement in gammaherpesvirus-driven carcinogenesis. Int. J. Mol. Sci..

[87] Lisek K., Campaner E., Ciani Y., Walerych D., Del Sal G. (2018). Mutant p53 tunes the nrf2-dependent antioxidant response to support survival of cancer cells. Oncotarget.

[88] Hamada S., Taguchi K., Masamune A., Yamamoto M., Shimosegawa T. (2017). Nrf2 promotes mutant k-ras/p53-driven pancreatic carcinogenesis. Carcinogenesis.

[89] Gilardini Montani M.S., Cecere N., Granato M., Romeo M.A., Falcinelli L., Ciciarelli U., D’Orazi G., Faggioni A., Cirone M. (2019). Mutant p53, stabilized by its interplay with hsp90, activates a positive feed-back loop between nrf2 and p62 that induces chemo-resistance to apigenin in pancreatic cancer cells. Cancers.

[90] Shin D., Kim E.H., Lee J., Roh J.L. (2018). Nrf2 inhibition reverses resistance to gpx4 inhibitor-induced ferroptosis in head and neck cancer. Free Radic. Biol. Med..

[91] Roh J.L., Kim E.H., Jang H., Shin D. (2017). Nrf2 inhibition reverses the resistance of cisplatin-resistant head and neck cancer cells to artesunate-induced ferroptosis. Redox Biol..

[92] Roh J.L., Jang H., Kim E.H., Shin D. (2017). Targeting of the glutathione, thioredoxin, and nrf2 antioxidant systems in head and neck cancer. Antioxid. Redox Signal..

[93] Jeong Y.I., Kim D.H., Chung K.D., Kim Y.H., Lee Y.S., Choi K.C. (2014). Antitumor activity of trigonelline-incorporated chitosan nanoparticles. J. Nanosci. Nanotechnol..

[94] Liao J.C., Lee K.T., You B.J., Lee C.L., Chang W.T., Wu Y.C., Lee H.Z. (2015). Raf/erk/nrf2 signaling pathway and mmp-7 expression involvement in the trigonelline-mediated inhibition of hepatocarcinoma cell migration. Food Nutr. Res..

[95] Sathiyaseelan A., Saravanakumar K., Jayalakshmi J., Gopi M., Shajahan A., Barathikannan K., Kalaichelvan P.T., Wang M.H. (2020). Trigonelline-loaded chitosan nanoparticles prompted antitumor activity on glioma cells and biocompatibility with pheochromocytoma cells. Int. J. Biol. Macromol..

[96] Sun T., Yu H.Y., Zhang C.L., Zhu T.N., Huang S.H. (2018). Respiratory syncytial virus infection up-regulates tlr7 expression by inducing oxidative stress via the nrf2/are pathway in a549 cells. Arch. Virol..

[97] Ozcelik B., Kartal M., Orhan I. (2011). Cytotoxicity, antiviral and antimicrobial activities of alkaloids, flavonoids, and phenolic acids. Pharm. Biol..

[98] Nguyen V., Zhang Y., Gao C., Cao X., Tian Y., Carver W., Kiaris H., Cui T., Tan W. (2022). The spike protein of SARS-CoV-2 impairs lipid metabolism and increases susceptibility to lipotoxicity: Implication for a role of nrf2. Cells.

[99] More G.K., Vervoort J., Steenkamp P.A., Prinsloo G. (2022). Metabolomic profile of medicinal plants with anti-rvfv activity. Heliyon.

[100] Gilardini Montani M.S., Santarelli R., Granato M., Gonnella R., Torrisi M.R., Faggioni A., Cirone M. (2019). Ebv reduces autophagy, intracellular ros and mitochondria to impair monocyte survival and differentiation. Autophagy.

[101] Gjyshi O., Bottero V., Veettil M.V., Dutta S., Singh V.V., Chikoti L., Chandran B. (2014). Kaposi’s sarcoma-associated herpesvirus induces nrf2 during de novo infection of endothelial cells to create a microenvironment conducive to infection. PLoS Pathog..

[102] Gjyshi O., Flaherty S., Veettil M.V., Johnson K.E., Chandran B., Bottero V. (2015). Kaposi’s sarcoma-associated herpesvirus induces nrf2 activation in latently infected endothelial cells through sqstm1 phosphorylation and interaction with polyubiquitinated keap1. J. Virol..

[103] Qin W., Guan D., Ma R., Yang R., Xing G., Shi H., Tang G., Li J., Lv H., Jiang Y. (2017). Effects of trigonelline inhibition of the nrf2 transcription factor in vitro on echinococcus granulosus. Acta Biochim. Biophys. Sin..

[104] Zhang W., Zhang Y., Chen S., Zhang H., Yuan M., Xiao L., Lu Y., Xu H. (2021). Trigonelline, an alkaloid from leonurus japonicus houtt., suppresses mast cell activation and ova-induced allergic asthma. Front. Pharmacol..

[105] Nugrahini A.D., Ishida M., Nakagawa T., Nishi K., Sugahara T. (2020). Trigonelline: An alkaloid with anti-degranulation properties. Mol. Immunol..

[106] Park H.J., Lee K.S., Lee E.K., Park N.C. (2018). Efficacy and safety of a mixed extract of trigonella foenum-graecum seed and lespedeza cuneata in the treatment of testosterone deficiency syndrome: A randomized, double-blind, placebo-controlled clinical trial. World J. Mens Health.

[107] Yoo G., Allred C.D. (2016). The estrogenic effect of trigonelline and 3,3-diindolymethane on cell growth in non-malignant colonocytes. Food Chem. Toxicol..

[108] Rathi A., Ishaq M., Najmi A.K., Akhtar M. (2020). Trigonelline demonstrated ameliorative effects in dexamethasone induced osteoporotic rats. Drug Res..

[109] Folwarczna J., Janas A., Pytlik M., Cegiela U., Sliwinski L., Krivosikova Z., Stefikova K., Gajdos M. (2016). Effects of trigonelline, an alkaloid present in coffee, on diabetes-induced disorders in the rat skeletal system. Nutrients.

[110] Zeng W.Y., Tan L., Han C., Zheng Z.Y., Wu G.S., Luo H.R., Li S.L. (2021). Trigonelline extends the lifespan of c. Elegans and delays the progression of age-related diseases by activating ampk, daf-16, and hsf-1. Oxid. Med. Cell. Longev..

[111] Bian L., Nguyen V.T., Tamaoki J., Endo Y., Dong G., Sato A., Kobayashi M. (2023). Genetic hyperactivation of nrf2 causes larval lethality in keap1a and keap1b-double-knockout zebrafish. Redox Biol..

[112] Nakhate K.T., Bharne A.P., Verma V.S., Aru D.N., Kokare D.M. (2018). Plumbagin ameliorates memory dysfunction in streptozotocin induced alzheimer’s disease via activation of nrf2/are pathway and inhibition of beta-secretase. Biomed. Pharmacother..

[113] Shaw P., Sen A., Mondal P., Dey Bhowmik A., Rath J., Chattopadhyay A. (2020). Shinorine ameliorates chromium induced toxicity in zebrafish hepatocytes through the facultative activation of nrf2-keap1-are pathway. Aquat. Toxicol..

[114] Vaglienti M.V., Subirada P.V., Joray M.B., Bonacci G., Sanchez M.C. (2023). Protective effect of no(2)-oa on oxidative stress, gliosis, and pro-angiogenic response in muller glial cells. Cells.

[115] Varshney V., Garabadu D. (2021). Ang(1–7) exerts nrf2-mediated neuroprotection against amyloid beta-induced cognitive deficits in rodents. Mol. Biol. Rep..

[116] Zhou Y.Q., Liu D.Q., Chen S.P., Chen N., Sun J., Wang X.M., Li D.Y., Tian Y.K., Ye D.W. (2020). Ppargamma activation mitigates mechanical allodynia in paclitaxel-induced neuropathic pain via induction of nrf2/ho-1 signaling pathway. Biomed. Pharmacother..

[117] Wang Y., Liao J., Luo Y., Li M., Su X., Yu B., Teng J., Wang H., Lv X. (2023). Berberine alleviates doxorubicin-induced myocardial injury and fibrosis by eliminating oxidative stress and mitochondrial damage via promoting nrf-2 pathway activation. Int. J. Mol. Sci..

[118] Eslami M., Esfandyari S., Aghahosseini M., Rashidi Z., Hosseinishental S.H., Brenjian S., Sobhani A., Amidi F. (2021). Astaxanthin protects human granulosa cells against oxidative stress through activation of nrf2/are pathway and its downstream phase ii enzymes. Cell J..

[119] Campolo M., Casili G., Biundo F., Crupi R., Cordaro M., Cuzzocrea S., Esposito E. (2017). The neuroprotective effect of dimethyl fumarate in an mptp-mouse model of parkinson’s disease: Involvement of reactive oxygen species/nuclear factor-kappab/nuclear transcription factor related to nf-e2. Antioxid. Redox Signal..

[120] Qin Q., Qu C., Niu T., Zang H., Qi L., Lyu L., Wang X., Nagarkatti M., Nagarkatti P., Janicki J.S. (2016). Nrf2-mediated cardiac maladaptive remodeling and dysfunction in a setting of autophagy insufficiency. Hypertension.

[121] Zang H., Wu W., Qi L., Tan W., Nagarkatti P., Nagarkatti M., Wang X., Cui T. (2020). Autophagy inhibition enables nrf2 to exaggerate the progression of diabetic cardiomyopathy in mice. Diabetes.

[122] Zang H., Mathew R.O., Cui T. (2020). The dark side of nrf2 in the heart. Front. Physiol..

[123] Desangles F. (1991). Diagnosis of fanconi’s anemia by nitrogen mustard induction of chromosome breakage in fibroblasts. Pathol. Biol..

